# Sexual violence against children and adolescents: analysis in peripheral contexts in the metropolis of São Paulo, Brazil

**DOI:** 10.1186/s13690-026-01933-z

**Published:** 2026-05-19

**Authors:** Carolina Lyriti Gomiero, Paula Hino, Girliani Silva de Sousa, Melina Aparecida Ferreira Iwamura, José Manuel Peixoto Caldas, Hugo Fernandes

**Affiliations:** 1 Department of Public Health, Federal University of São Paulo, São Paulo, São Paulo Brazil; 2 Community of Madrid, Fundación Carolina, Madrid, Spain

**Keywords:** Sex Offenses, Child Abuse, Sexual, Adolescent, Social Vulnerability

## Abstract

**Background:**

Although sexual violence against children and adolescents is a global public health imperative, its manifestation in Brazil reveals a critical gap between international protocols and the socioeconomic complexity of urban peripheries. The present study aimed to analyze the characteristics of sexual violence against children and adolescents living in the outskirts of São Paulo, Brazil.

**Method:**

A cross-sectional study with data from mandatory reports of sexual violence, extracted from the mandatory notification forms of the Notifiable Diseases Information System (SINAN), occurring between 2016 and 2024. The sample consisted of victims aged between 1 and 17 years, residing in 23 administrative districts considered to be on the outskirts of the city of São Paulo. Univariate statistical analysis and the chi-square test were applied to verify possible relationships between types of violence and variables with a significance level of 5% (alpha = 0.05). A total of 21,576 cases of sexual violence were identified during the period outlined. Female victims were most common, concentrated in the 11–14 age group. Rape was the most prevalent type of violence, accounting for 87.8% of cases involving female victims and 85.5% of cases involving male victims.

**Results:**

The home was the most frequent location for these types of violence. Sexual harassment, in particular, occurred mainly at home (76.6%) and at school (84.7%). The likely perpetrator was predominantly male. The home was the most frequent location for these types of violence. Sexual harassment, in particular, occurred mainly at home (76.6%) and at school (84.7%). The likely perpetrator was predominantly male and in the adult age group (25–59 years). The father was the most frequent perpetrator of sexual harassment (80.8%), and the boyfriend/intimate partner was the most frequent perpetrator of rape (71.9%) in cases involving a relationship. Rape was reported in 92.7% of cases involving pregnant women. Victims with disabilities suffered more sexual harassment (69.3%) than rape (23.8%). The predominant motive was sexism. Suspicious alcohol consumption by the perpetrator was a trend in rape cases.

**Conclusions:**

The study indicates that the types of sexual violence in the outskirts of São Paulo are marked by a predominance of younger female adolescent victims and a high prevalence of rape within the family environment. The findings reinforce the urgency of interventions to identify forms of sexual violence in public health services to ensure the comprehensive protection of this doubly vulnerable group. The high number of blank or incomplete fields in the notification forms suggests a need for training and awareness among the professionals who fill them out.

**Table:** 

Text box 1. Contributions to the literature
• Tens of thousands of cases of sexual violence in the suburbs of São Paulo, mainly affecting girls and concentrated in the transition to adolescence.
• Rape has become the most recurrent form of violence among victims of both sexes, with a critical incidence even among young children.
• The family home and school are the most common locations for assaults, which are mostly committed by adult men with close ties, such as fathers and intimate partners.
• Groups in situations of greater vulnerability, such as pregnant women and people with intellectual disabilities, suffer specific forms of violence, with rape being predominant in the first group and harassment in the second.
• The study points to serious flaws in the completion of notifications, which demonstrates the need for improvements in information systems so that public protection policies are more effective.

## Background

Sexual violence is defined by the World Health Organization (WHO) as any unwanted sexual act, attempted sexual act, or sexual advances, as well as actions to commercialize or use a person’s sexuality through coercion. The central element of this definition is the absence of free and informed consent, with coercion being the primary tool of the aggressor, which can manifest itself through physical force, psychological intimidation, or taking advantage of the victim’s vulnerability (such as inability to react, intoxication, or age). Therefore, sexual violence should not be understood as an act of passion or uncontrolled desire, but rather as a manifestation of power, control, and domination [1]. 

This form of violence takes many forms, going beyond the strictest concept of rape. It encompasses sexual harassment, which seeks to humiliate and intimidate; sexual abuse, which exploits the hierarchy of power for the aggressor’s satisfaction; sexual exploitation, which commodifies the sexuality of victims, as in the case of forced prostitution, among other forms. Such acts are universal and affect people of all ages, genders, and social contexts. However, children and adolescents are more vulnerable than people in other age groups because they do not always recognize the occurrence as an act of violence, trust or depend on the aggressor, and are easily discouraged from reporting victimization with threats and other aggression. [1, 2]

Researchers cite that sexual violence against children and adolescents manifests itself in three main areas: intrafamilial abuse, which is the most common and hidden, perpetrated by trusted individuals within the home; abuse outside the family, committed by individuals in positions of authority or outside the inner circle; and commercial sexual exploitation, which turns the body of the child or adolescent into a commodity for the profit of third parties [3–5]. 

It is estimated that more than one billion children suffer some form of violence annually, and sexual violence is one of the most serious forms within this scope. Global statistics from the United Nations Children’s Fund (UNICEF) indicate that, in total, nearly 90 million living boys and girls have experienced sexual violence [5]. With regard to gender, the proportion is alarming: globally, about one in five girls and women alive today have been victims of sexual violence in childhood, while the estimate for boys and men alive today is approximately one in seven. [5, 6]

In urban peripheries and pockets of poverty, sexual violence against children and adolescents is intensified by multilevel vulnerability, such as precarious housing (overcrowding, lack of privacy), which facilitates intra-family abuse, and the fragility of institutional protection networks. The absence of safe spaces and exposure to armed violence and organized crime increase the risk of grooming and commercial sexual exploitation (CSE), often motivated by economic necessity and facilitated by the promise of goods. In these contexts, fear of retaliation and the fact that the perpetrator is often the family provider promote silence, while geographical barriers and lack of resources (transportation) hinder access to specialized health, justice, and psychological support services, perpetuating the cycle of revictimization and impunity [2–6]. 

Whether in the short or long term, the repercussions of sexual violence in childhood and adolescence are serious and complex. Physical injuries, sexually transmitted infections (STIs), and, in the case of adolescents, early pregnancy may occur. However, the psychological impact is the most lasting, manifesting itself as post-traumatic stress disorder (PTSD), chronic depression, anxiety, non-suicidal self-harm, learning problems, aggressive behavior, and difficulties in establishing healthy interpersonal relationships. [2, 3]

A review study conducted by Canadian researchers pointed out that there are different types of geographical and economic barriers that influence the intersectoral response to sexual violence in central and peripheral regions. These barriers may include the distance from the place of residence to health and social protection services, areas controlled by parallel criminal powers (e.g., drug trafficking), the absence of social income programs for children and adolescents, and even a lack of information on the subject and human rights. However, it mentions that statistical data on peripheral or suburban populations are still scarce in the scientific literature and that this may directly affect the planning of measures for prevention, control, and assistance to those most vulnerable [7]. 

Sexual violence against children and adolescents in Brazilian suburbs highlights the discrepancy between global public health guidelines and the reality of a country where 80.7% of victims of sexual exploitation and abuse are children and adolescents [8]. This scenario is aggravated by the structural precariousness of these territories, where the absence of state protection mechanisms transforms socioeconomic vulnerability into a chronic risk factor, challenging the effectiveness of universal intervention models. São Paulo is the largest city in Latin America, with more than 11 million inhabitants, reaching 19 million annually when considering the floating population (tourists, seasonal or daily workers, students, merchants, congressmen, and the like), providing great cultural, social, and financial diversity. [8, 9] Its population ends up being representative of the entire country, as a mixture of the culture of each region.

This study is justified by the need to expose how sexual violence against children and adolescents in Brazilian suburbs is exacerbated by socio-territorial exclusion that compromises the comprehensive protection provided for by law. The problem possibly lies in the State’s difficulty in extending comprehensive health policies to areas marked by deep socioeconomic divisions, where the lack of care makes children’s bodies more vulnerable. In this sense, the hypothesis is established that sexual violence in these contexts does not affect the population homogeneously, with younger children and those living in neighborhoods with higher poverty rates being the main victims, since the intersection between dependence on adult care and the fragility of community support networks increases exposure to risk and the invisibility of abuse. More importantly, the research results may provide empirical bases and essential strategic guidelines for authorities and protection networks (health, education, social assistance, human rights, and justice) to develop and implement focused, effective, and territorially adapted public policies aimed at prevention, intervention, and comprehensive protection of this doubly vulnerable group. Thus, the present study aimed to analyze the characteristics of sexual violence against children and adolescents living in the outskirts of the city of São Paulo, Brazil.

## Method

### Study design

This was an epidemiological study with a cross-sectional design. Methodological quality was established to ensure that the study design and reporting were free of bias. Thus, the instrument used to conduct the study was Strengthening the Reporting of Observational studies in Epidemiology (STROBE) [10] which is a guide consisting of a checklist of 22 essential items that must be reported in observational studies, such as cohort, case-control, and cross-sectional studies. In terms of bias measurement, it functions as a transparency tool, allowing readers to judge the reliability of the data based on the clarity of the limitations presented.

### Data collection, location, and period

Data were collected from the information contained in the Notification Forms for Suspected or Confirmed Cases of Sexual Violence from the Notifiable Diseases Information System (SINAN). These forms are standardized by the Brazilian Ministry of Health and adopted throughout the country. The variables studied regarding victims and incidents are mostly classified as nominal categorical variables. including gender (of the victim and perpetrator), race/color, administrative district, sexual orientation, gender identity, pregnancy, presence and type of disability, as well as details of the event such as location, motivation, means of aggression, relationship with the victim, suspected alcohol use, and health procedures performed. The ordinal variables investigated were age group (both of the victim and the aggressor), level of education, gestational age (in trimesters), and the number of people involved in the incident.

All data were extracted from TabNet, a program developed by the Department of Informatics of the Unified Health System (DATASUS) and made available on the internet without access restrictions. These data were entered into the Notifiable Diseases Information System (SINAN) by reporting health units, such as Health Surveillance Units, Hospitals, outpatient clinics, and other general or specialized public health services.

The city of São Paulo was selected because it makes notifications available in the TabNet system for public consultation in a more agile manner, in contrast to the national system, considering the different dimensions and particularities of access to SINAN by states and municipalities. In addition. The period defined for the research covered violence records from 2016 to 2024. Data prior to 2016 were not collected because the mandatory notification form for cases of violence underwent significant changes at the end of 2015. The data were collected between November and December 2025, and tabulation was performed using Excel 365 software. Data extraction from the Tabnet system was performed by two researchers simultaneously to avoid errors in collection. No discrepancies were found in any variable. Subsequently, a third researcher (external to the project) checked the data to verify the security of the information.

### Inclusion and exclusion criteria

Reports of sexual violence (harassment, rape, child pornography, sexual exploitation, and other similar but unspecified acts) were included for victims aged > 1 year and up to 17 years, residing in administrative districts considered peripheral by the Municipal Secretariat for Coordination of Sub-Prefectures [9], which considers aspects such as distance from the city center, geographical boundaries (such as rivers, parks, and avenues), and population density. Thus, the selected administrative districts were: Itaim Paulista, Guaianases, Cidade Tiradentes, Itaquera, São Matheus, Sapopemba, Aricanduva-Formosa, Penha, Tremembé-Jaçanã, Santana, Vila Maria, Casa Verde, Perus, Pirituba, Freguesia do Ó, Butantã, Campo Limpo, M’Boi Mirim, Parelheiros, Capela do Socorro, Santo Amaro, Cidade Ademar, and Jabaquara. The age of 17 is justified because it is classified as bordering adulthood by SINAN. Notifications of children and adolescents residing in central districts, such as Lapa, Sé, Pinheiro, Vila Mariana, and Ipiranga, as well as those whose territory is considered central, such as Mooca and Vila Prudente, were excluded. Notifications of suspected or confirmed cases of self-inflicted violence, physical (non-sexual), psychological, torture, financial, human trafficking, neglect, and legal intervention were also excluded.

Thus, the sample obtained consisted of 21,576 reports of suspected or confirmed cases of sexual violence against children and adolescents residing in peripheral regions of São Paulo during the period described.

### Analysis

The data were organized and formatted using Excel 365. Next, a univariate statistical analysis was performed using R software, version 4.0.2, supplemented by a descriptive analysis based on absolute frequencies and percentages. The chi-square test was used to verify the related between the types of sexual violence and the variables, considering a significance level of 5% (alpha = 0.05). Due to the nature of data tabulation by the TabNet information system of the Unified Health System, which does not provide individual information for each notification, it was not possible to perform the necessary adjustment procedures for logistic regression, data imputation, or multivariate analysis. For ecological comparisons, standardized proportions, or stratified analyses with effect size reports, it would be advisable to make individualized data available (on a case-by-case basis).

## Results

Analysis of victim data revealed a possible relationship with variables related to age group, pregnancy, educational level, peripheral administrative district (residence), and presence of disability (*p* < .0001), as well as sexual orientation (*p* = .0317) and gender identity (*p* = .0009), as shown in Table 1.

**Table 1:** Distribution of sociodemographic characteristics of children and adolescents who are victims of sexual violence, São Paulo, São Paulo, 2025

VARIABLE	TYPE OF SEXUAL VIOLENCE					*p* - value
	HARASSMENT	RAPE	CHILD PORNOGRAPHY	SEXUAL EXPLOITATION	OTHERS	
Age group (SINAN)						
> 1 year	44 (9.6%)	405 (88.8%)	4 (0.9%)	3 (0.7%)	0 (0%)	< 0.0001
1–3 years	343 (11%)	2741 (87.7%)	14 (0.4%)	22 (0.7%)	7 (0.2%)	
4–5 years	196 (7.1%)	2506 (91.1%)	27 (1%)	17 (0.6%)	4 (0.1%)	
6–10 years	692 (12.3%)	4804 (85.5%)	74 (1.3%)	42 (0.7%)	6 (0.1%)	
11–14 years	741 (11.7%)	5497 (86.5%)	43 (0.7%)	66 (1%)	10 (0.2%)	
15–17 years	344 (10.5%)	2888 (88.4%)	9 (0.3%)	25 (0.8%)	2 (0.1%)	
Gender						
Female	1.650 (11.2%)	12.950 (87.8%)	120 (0.8%)	110 (0.7%)	20 (0.1%)	< 0.0001
Male	680 (10.5%)	5.550 (85.5%)	50 (0.8%)	60 (0.9%)	8 (0.1%)	
Ignored/Blank	30 (8.5%)	341 (89.5%)	1 (0.3%)	5 (1.3%)	1 (0.3%)	
Race/color						
White	1158 (11.9%)	8367 (86%)	75 (0.8%)	115 (1.2%)	17 (0.2%)	0.3947
Brown	1059 (12.3%)	7353 (85.6%)	74 (0.9%)	89 (1%)	18 (0.2%)	
Black	292 (13.3%)	1856 (84.4%)	21 (1%)	26 (1.2%)	4 (0.2%)	
Indigenous	34 (12.9%)	227 (86%)	2 (0.8%)	1 (0.4%)	0 (0%)	
Yellow	23 (12.3%)	157 (84%)	1 (0.5%)	4 (2.1%)	2 (1.1%)	
Ignored	107 (11.4%)	800 (84.9%)	11 (1.2%)	20 (2.1%)	4 (0.4%)	
Pregnant						
Yes	25 (3.8%)	608 (92.7%)	11 (1.7%)	8 (1.2%)	4 (0.6%)	< 0.0001
No	676 (12.4%)	4631 (84.8%)	38 (0.7%)	98 (1.8%)	19 (0.3%)	
Ignored	106 (7.8%)	1218 (89.8%)	7 (0.5%)	24 (1.8%)	2 (0.1%)	
Does not apply	1872 (12.8%)	12,484 (85.3%)	135 (0.9%)	127 (0.9%)	20 (0.1%)	
Gestational age (if pregnant)						
First trimester	16 (5.4%)	271 (92.2%)	2 (0.7%)	4 (1.4%)	1 (0.3%)	0.9419
Second trimester	5 (3.7%)	126 (92.6%)	1 (0.7%)	2 (1.5%)	2 (1.5%)	
Third trimester	3 (3.9%)	70 (92.1%)	1 (1.3%)	1 (1.3%)	1 (1.3%)	
Ignored	1 (1.9%)	41 (78.8%)	7 (13.5%)	1 (1.9%)	2 (3.8%)	
Education						
Illiterate	21 (17.2%)	94 (77%)	4 (3.3%)	3 (2.5%)	0 (0%)	< 0.0001
1st to 4th grade incomplete	324 (14.8%)	1801 (82.5%)	28 (1.3%)	26 (1.2%)	5 (0.2%)	
4th grade complete	85 (9%)	838 (88.7%)	12 (1.3%)	8 (0.8%)	2 (0.2%)	
5th to 8th grade incomplete	2072 (34.1%)	3910 (64.3%)	43 (0.7%)	49 (0.8%)	9 (0.1%)	
Elementary school complete	292 (31%)	633 (67.1%)	1 (0.1%)	15 (1.6%)	2 (0.2%)	
High school incomplete	875 (32.6%)	1785 (66.4%)	7 (0.3%)	19 (0.7%)	2 (0.1%)	
High school complete	176 (24.8%)	494 (69.7%)	8 (1.1%)	27 (3.8%)	4 (0.6%)	
Higher education incomplete	8 (16.7%)	32 (66.7%)	1 (2.1%)	7 (14.6%)	0 (0%)	
Higher education complete	8 (16.7%)	18 (37.5%)	15 (31.3%)	5 (10.4%)	2 (4.2%)	
Ign/Blank	309 (14.2%)	1805 (82.8%)	21 (1%)	39 (1.8%)	6 (0.3%)	
Peripheral Administrative District (residence)						
Campo Limpo	294 (78.8%)	32 (8.6%)	29 (7.8%)	16 (4.3%)	2 (0.5%)	< 0.0001
M’Boi Mirim	-	-	-	-	-	
Parelheiros	247 (74.8%)	49 (14.8%)	6 (1.8%)	28 (8.5%)	0 (0%)	
Capela do Socorro	-	-	-	-	-	
Santo Amaro	22 (55%)	3 (7.5%)	4 (10%)	11 (27.5%)	0 (0%)	
Cidade Ademar	240 (75.9%)	41 (13%)	17 (5.4%)	15 (4.7%)	3 (0.9%)	
Jabaquara	63 (63.6%)	17 (17.2%)	7 (7.1%)	9 (9.1%)	3 (3%)	
Tremembé-Jaçanã	276 (73.4%)	58 (15.4%)	18 (4.8%)	21 (5.6%)	3 (0.8%)	
Santana	17 (54.8%)	6 (19.4%)	4 (12.9%)	3 (9.7%)	1 (3.2%)	
Vila Maria	69 (68.3%)	21 (20.8%)	5 (5%)	5 (5%)	1 (1%)	
Casa Verde	119 (82.1%)	20 (13.8%)	1 (0.7%)	5 (3.4%)	0 (0%)	
Perus	84 (65.6%)	26 (20.3%)	4 (3.1%)	11 (8.6%)	3 (2.3%)	
Pirituba	119 (73.9%)	30 (18.6%)	5 (3.1%)	7 (4.3%)	0 (0%)	
Freguesia do Ó	29 (56.9%)	9 (17.6%)	4 (7.8%)	8 (15.7%)	1 (2%)	
Butantã	11 (61.1%)	0 (0%)	5 (27.8%)	2 (11.1%)	0 (0%)	
Itaim Paulista	375 (75.8%)	59 (11.9%)	27 (5.5%)	24 (4.8%)	10 (2%)	
Guaianases	109 (80.1%)	8 (5.9%)	4 (2.9%)	13 (9.6%)	2 (1.5%)	
Cidade Tiradentes	192 (73.3%)	36 (13.7%)	16 (6.1%)	13 (5%)	5 (1.9%)	
Itaquera	156 (69.3%)	40 (17.8%)	12 (5.3%)	12 (5.3%)	5 (2.2%)	
São Matheus	76 (66.1%)	24 (20.9%)	3 (2.6%)	10 (8.7%)	2 (1.7%)	
Sapopemba	182 (65%)	55 (19.6%)	9 (3.2%)	33 (11.8%)	1 (0.4%)	
Aricanduva-Formosa	32 (48.5%)	21 (31.8%)	6 (9.1%)	4 (6.1%)	3 (4.5%)	
Penha	55 (66.3%)	17 (20.5%)	5 (6%)	6 (7.2%)	0 (0%)	
Sexual orientation						
Heterosexual	1977 (28.4%)	4845 (69.5%)	33 (0.5%)	102 (1.5%)	15 (0.2%)	0.0317
Homosexual	104 (30.7%)	224 (66.1%)	2 (0.6%)	8 (2.4%)	1 (0.3%)	
Bisexual	123 (35.7%)	209 (60.6%)	3 (0.9%)	8 (2.3%)	2 (0.6%)	
Not applicable	6349 (35.3%)	11,377 (63.3%)	123 (0.7%)	97 (0.5%)	19 (0.1%)	
Ignored	1103 (32.8%)	2186 (65%)	23 (0.7%)	41 (1.2%)	8 (0.2%)	
Gender identity						
Transvestite	5 (29.4%)	9 (52.9%)	3 (17.6%)	0 (0%)	0 (0%)	0.0009
Transsexual woman	51 (21.6%)	177 (75%)	2 (0.8%)	5 (2.1%)	1 (0.4%)	
Transsexual man	28 (31.8%)	54 (61.4%)	3 (3.4%)	3 (3.4%)	0 (0%)	
Not applicable	8356 (34.8%)	15,253 (63.6%)	155 (0.6%)	195 (0.8%)	34 (0.1%)	
Ignored	1216 (26.1%)	3348 (71.9%)	27 (0.6%)	53 (1.1%)	10 (0.2%)	
Presence of Disability						
Yes	667 (69.3%)	229 (23.8%)	22 (2.3%)	37 (3.8%)	7 (0.7%)	< 0.0001
No	7859 (73.6%)	2459 (23%)	142 (1.3%)	187 (1.8%)	31 (0.3%)	
Ignored	1096 (60.5%)	658 (36.3%)	20 (1.1%)	31 (1.7%)	7 (0.4%)	
Type of disability or disorder						
Physical disability	45 (80.4%)	8 (14.3%)	1 (1.8%)	2 (3.6%)	0 (0%)	0.5958
Intellectual disability	222 (71.6%)	70 (22.6%)	5 (1.6%)	11 (3.5%)	2 (0.6%)	
Visual impairment	29 (74.4%)	7 (17.9%)	2 (5.1%)	1 (2.6%)	0 (0%)	
Hearing impairment	18 (75%)	5 (20.8%)	1 (4.2%)	0 (0%)	0 (0%)	
Mental disorder	242 (68.2%)	74 (20.8%)	13 (3.7%)	22 (6.2%)	4 (1.1%)	
Behavioral disorder	194 (70.3%)	60 (21.7%)	8 (2.9%)	9 (3.3%)	5 (1.8%)	
Other	107 (66.9%)	45 (28.1%)	4 (2.5%)	3 (1.9%)	1 (0.6%)	

The sociodemographic profile of victims of sexual violence in São Paulo shows a predominance of assaults against females, who accounted for 68.4% of reported cases. In both sexes, rape was the main type of violence, affecting 87.8% of women and 85.5% of men. With regard to age group, although the highest absolute number of reports occurred among adolescents aged 11 to 14 years, a proportionally higher vulnerability to rape was observed in the group of children aged 4 to 5 years, in which this type of violence accounted for 91.1% of occurrences. These variations in the distribution of types of violence according to age, sex, and gestational status were statistically significant (*p* < .0001).

Regarding race/color and education markers, the victims were mainly concentrated in the white (45.1%) and brown (39.8%) groups. In terms of formal education, incomplete elementary school was the most prevalent. However, an atypical finding occurred among victims with complete higher education, where the incidence of child pornography was substantially higher (31.3%) when compared to other levels of education. Geographically, violence was most concentrated in the outlying administrative districts, particularly Itaim Paulista, Tremembé-Jaçanã, and Campo Limpo, which together accounted for almost one-third of the reports with an identified location.

The analysis of specific identities and vulnerabilities revealed that most victims identified as LGBTQIA+ declared themselves to be heterosexual (91.1%). However, among gender-nonconforming identities, transgender women were the most affected (69.2%), with a high prevalence of rape (75%). In the group of people with disabilities, intellectual disability was the most common. Unlike the general pattern of the sample, this subgroup was mostly victimized by sexual harassment (69.3%) rather than rape. It should be noted that the high incidence of missing or blank data in critical variables limits the comprehensive analysis of the phenomenon, as well as in Table 2, which presents the characteristics of the incidents and the perpetrator, according to the type of violence.

**Table 2:** Distribution of characteristics of incidents and perpetrators of sexual violence against children and adolescents, São Paulo, 2025

Variable	Type of sexual violence					*p*-value
	Harassment	Rape	Child pornography	Sexual exploitation	Other	
Location of the incident						
Residence	6796 (76,6%)	1779 (20%)	136 (1,5%)	137 (1,5%)	26 (0,3%)	< .0001
Multi-unit housing	98 (67,1%)	34 (23,3%)	4 (2,7%)	8 (5,5%)	2 (1,4%)	
School	531 (84,7%)	80 (12,8%)	6 (1%)	8 (1,3%)	2 (0,3%)	
Sports facility	31 (70,5%)	11 (25%)	1 (2,3%)	1 (2,3%)	0 (0%)	
Bar or similar establishment	50 (52,1%)	41 (42,7%)	2 (2,1%)	3 (3,1%)	0 (0%)	
Public street	376 (50,3%)	337 (45,1%)	6 (0,8%)	29 (3,9%)	0 (0%)	
Retail/Services	70 (66,7%)	30 (28,6%)	1 (1%)	4 (3,8%)	0 (0%)	
Industrial/Construction	10 (66,7%)	4 (26,7%)	1 (6,7%)	0 (0%)	0 (0%)	
Other	902 (73,1%)	275 (22,3%)	17 (1,4%)	32 (2,6%)	8 (0,6%)	
Reason						
Sexism	2858 (58,1%)	1951 (39,7%)	40 (0,8%)	65 (1,3%)	3 (0,1%)	< .0001
Homophobia/Biphobia/Transphobia	54 (76,1%)	11 (15,5%)	3 (4,2%)	3 (4,2%)	0 (0%)	
Racism	2 (50%)	0 (0%)	2 (50%)	0 (0%)	0 (0%)	
Religious intolerance	3 (100%)	0 (0%)	0 (0%)	0 (0%)	0 (0%)	
Xenophobia	1 (100%)	0 (0%)	0 (0%)	0 (0%)	0 (0%)	
Generational conflict	141 (82,5%)	21 (12,3%)	2 (1,2%)	7 (4,1%)	0 (0%)	
Homelessness	19 (52,8%)	8 (22,2%)	1 (2,8%)	8 (22,2%)	0 (0%)	
Disability	71 (87,7%)	9 (11,1%)	1 (1,2%)	0 (0%)	0 (0%)	
Other	2031 (83,1%)	282 (11,5%)	44 (1,8%)	72 (2,9%)	15 (0,6%)	
Not applicable	1967 (86,6%)	209 (9,2%)	48 (2,1%)	36 (1,6%)	12 (0,5%)	
Not specified	2509 (71,8%)	857 (24,5%)	48 (1,4%)	65 (1,9%)	15 (0,4%)	
Means of assault						
Physical force/beating	3140 (70,8%)	1114 (25,1%)	54 (1,2%)	107 (2,4%)	19 (0,4%)	< .0001
Hanging	86 (67,7%)	25 (19,7%)	5 (3,9%)	10 (7,9%)	1 (0,8%)	
Blunt object	52 (65%)	14 (17,5%)	3 (3,8%)	8 (10%)	3 (3,8%)	
Sharp object	111 (71,6%)	28 (18,1%)	4 (2,6%)	8 (5,2%)	4 (2,6%)	
Hot substance/object	23 (56,1%)	9 (22%)	2 (4,9%)	6 (14,6%)	1 (2,4%)	
Poisoning/intoxication	49 (46,7%)	40 (38,1%)	1 (1%)	9 (8,6%)	6 (5,7%)	
Firearm	43 (58,9%)	25 (34,2%)	1 (1,4%)	3 (4,1%)	1 (1,4%)	
Threat	2126 (76,6%)	515 (18,5%)	56 (2%)	70 (2,5%)	10 (0,4%)	
Other	1679 (78,8%)	315 (14,8%)	57 (2,7%)	63 (3%)	16 (0,8%)	
Number of people involved						
One	7766 (71,1%)	2813 (25,8%)	132 (1,2%)	179 (1,6%)	28 (0,3%)	< .0001
Two or more	1450 (74,3%)	393 (20,1%)	44 (2,3%)	53 (2,7%)	12 (0,6%)	
Unknown	410 (70,8%)	138 (23,8%)	6 (1%)	20 (3,5%)	5 (0,9%)	
Relationship/degree of kinship						
Father	1695 (80,8%)	328 (15,6%)	38 (1,8%)	26 (1,2%)	10 (0,5%)	< .0001
Mother	389 (84,6%)	32 (7%)	22 (4,8%)	12 (2,6%)	5 (1,1%)	
Stepfather	1465 (73,5%)	482 (24,2%)	19 (1%)	21 (1,1%)	5 (0,3%)	
Stepmother	45 (88,2%)	3 (5,9%)	1 (2%)	1 (2%)	1 (2%)	
Spouse	48 (64%)	6 (8%)	1 (1,3%)	16 (21,3%)	4 (5,3%)	
Ex-spouse	46 (74,2%)	2 (3,2%)	1 (1,6%)	10 (16,1%)	3 (4,8%)	
Boyfriend/girlfriend	75 (40,1%)	98 (52,4%)	1 (0,5%)	10 (5,3%)	3 (1,6%)	
Ex-boyfriend/girlfriend	44 (59,5%)	26 (35,1%)	0 (0%)	4 (5,4%)	0 (0%)	
Son/daughter	50 (98%)	0 (0%)	0 (0%)	0 (0%)	1 (2%)	
Brother/sister	431 (81,2%)	79 (14,9%)	12 (2,3%)	8 (1,5%)	1 (0,2%)	
Friend/acquaintance	2865 (70,8%)	1057 (26,1%)	54 (1,3%)	59 (1,5%)	11 (0,3%)	
Stranger	890 (50,5%)	784 (44,5%)	26 (1,5%)	56 (3,2%)	7 (0,4%)	
Caregiver	158 (90,3%)	9 (5,1%)	5 (2,9%)	2 (1,1%)	1 (0,6%)	
Employer/boss	29 (85,3%)	3 (8,8%)	0 (0%)	2 (5,9%)	0 (0%)	
Person with an institutional relationship	51 (59,3%)	24 (27,9%)	3 (3,5%)	6 (7%)	2 (2,3%)	
Police officer/law enforcement officer	8 (72,7%)	1 (9,1%)	1 (9,1%)	1 (9,1%)	0 (0%)	
Self	14 (43,8%)	12 (37,5%)	0 (0%)	1 (3,1%)	5 (15,6%)	
Other	623 (54,5%)	436 (38,1%)	35 (3,1%)	40 (3,5%)	9 (0,8%)	
Gender of the likely perpetrator						
Male	2259 (39%)	3139 (54,2%)	147 (2,5%)	218 (3,8%)	25 (0,4%)	< .0001
Female	109 (61,6%)	37 (20,9%)	10 (5,6%)	14 (7,9%)	7 (4%)	
Both genders	206 (73%)	40 (14,2%)	20 (7,1%)	9 (3,2%)	7 (2,5%)	
Unknown	105 (40,2%)	131 (50,2%)	6 (2,3%)	13 (5%)	6 (2,3%)	
Age group of the likely perpetrator						
Child (0–9 years)	83 (79,8%)	12 (11,5%)	8 (7,7%)	1 (1%)	0 (0%)	< .0001
Adolescent (10–19 years)	479 (42,4%)	597 (52,8%)	22 (1,9%)	27 (2,4%)	5 (0,4%)	
Young adult (20–24 years)	199 (32,1%)	365 (59%)	22 (3,6%)	30 (4,8%)	3 (0,5%)	
Adult (25–59 years)	1392 (43,4%)	1553 (48,4%)	96 (3%)	139 (4,3%)	26 (0,8%)	
Older adult (60 years or older)	139 (54,5%)	93 (36,5%)	7 (2,7%)	16 (6,3%)	0 (0%)	
Unknown	387 (32,3%)	728 (60,8%)	29 (2,4%)	43 (3,6%)	11 (0,9%)	

All variables analyzed regarding the characteristics of the incidents and perpetrators were statistically significant (*p* < .0001). The home was the main location of occurrence, accounting for the majority of cases, including 76.6% of harassment and 20% of rape. School stood out as the second setting with the highest concentration of sexual harassment (84.7%).

With regard to motivation and methods, sexism was the most frequent cause, identified in 58.1% of harassment cases and 39.7% of rape cases. The dynamics of violence primarily involved the use of physical force or beating (70.8% in harassment; 25.1% in rape), in addition to recurring threats. In the vast majority of cases (71.6%), the violence was perpetrated by a single individual.

As for the profile of the aggressor, men predominated (present in 54.2% of rape cases) and were predominantly adults between the ages of 25 and 59. There was a significant variation in the relationship with the victim: while the father was the most frequent perpetrator in situations of harassment (80.8%), the boyfriend was the main perpetrator related with rape (52.4%). Friends and acquaintances also represented a significant proportion, especially in cases of harassment (70.8%).

**Fig. 1:**
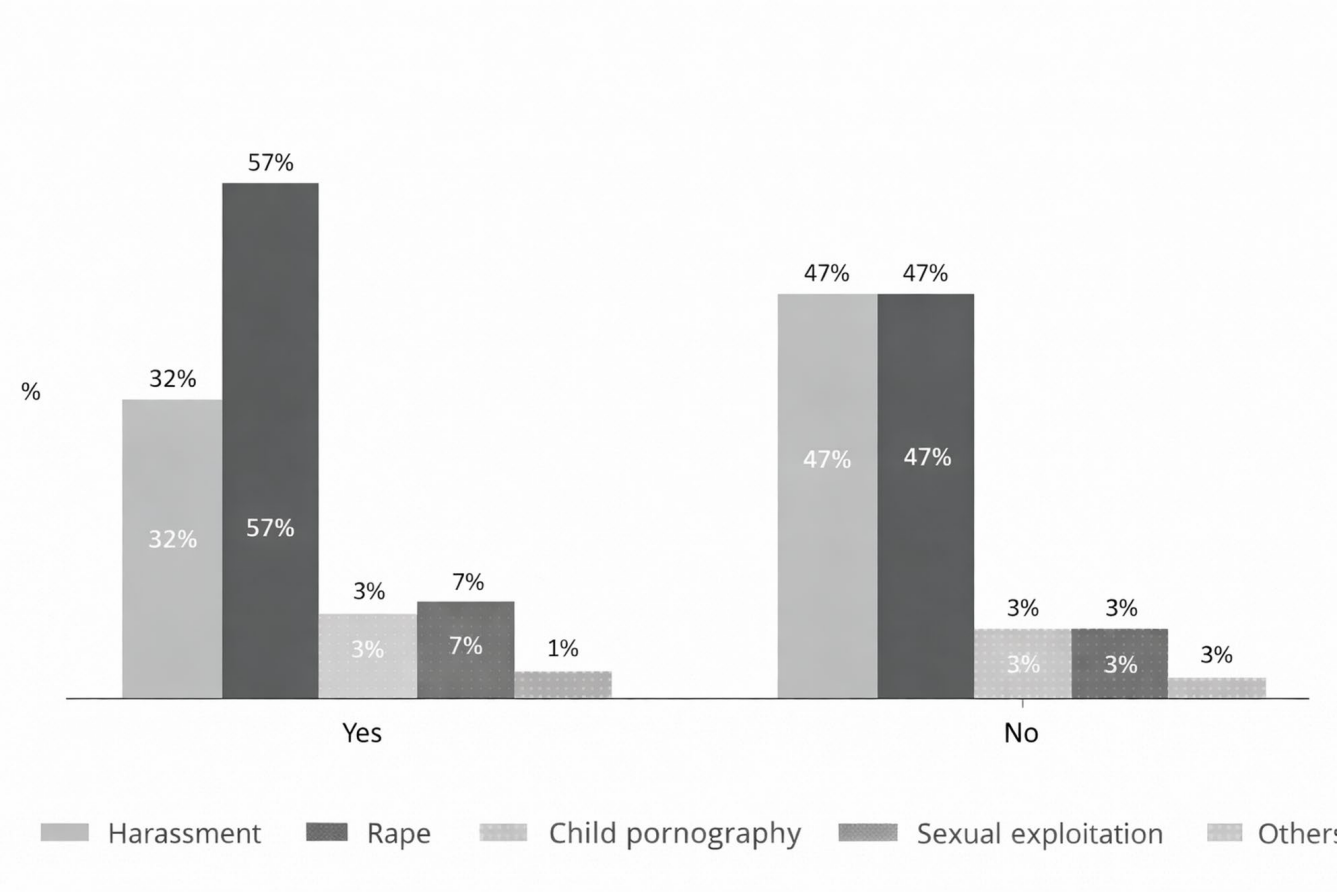
Suspected alcohol use by the perpetrator of sexual violence at the time of the incident, São Paulo, São Paulo, 2025

Figure 1 shows the percentage distribution of different types of sexual violence (harassment, rape, child pornography, sexual exploitation, and others) stratified by whether or not the perpetrator was suspected of alcohol use at the time of the incident.

Figure 1 shows that in cases where alcohol use by the perpetrator is suspected, there is a concentration of rape cases, which account for 57% of occurrences. Harassment is the second most prevalent type, with 32%, resulting in a combined proportion of 89% for these two categories. The remaining types of violence, such as child pornography, sexual exploitation, and others, show a low related with suspected alcohol consumption. This pattern suggests that the presence of alcohol consumption in the context of assault is related with a higher probability of rape.

**Fig. 2:**
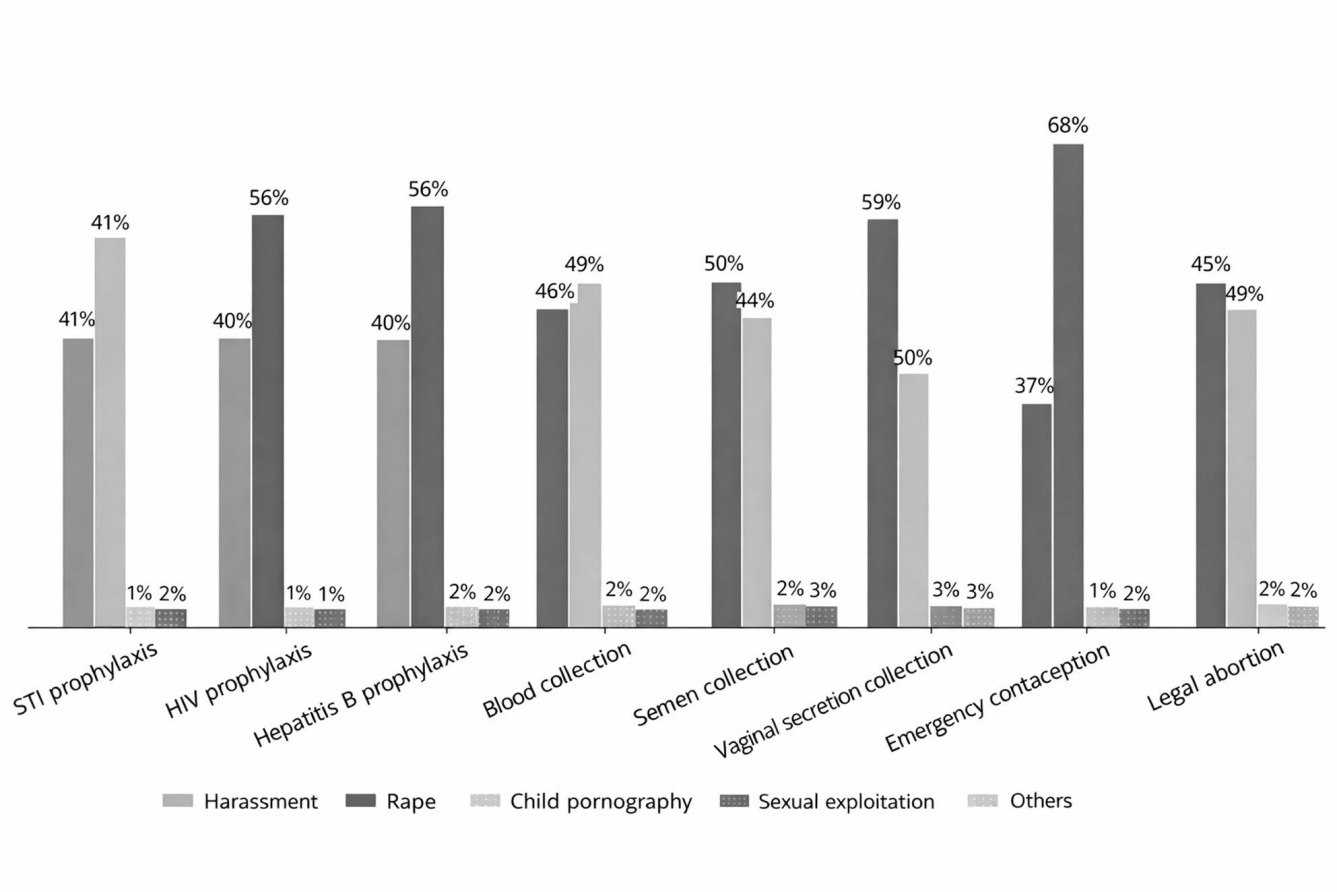
Procedures performed by health professionals according to the type of sexual violence, São Paulo, São Paulo, 2025

The main procedures performed by health professionals in the care of children and adolescents who are victims of sexual violence are shown in Fig. 2.

Figure 2 illustrates the percentage distribution of different types of violence, classified as harassment, rape, child pornography, sexual exploitation, and others, in relation to categories of health procedures or intervention contexts. There is a marked predominance of the categories of rape and harassment over the others, which consistently show marginal proportions (generally between 0 and 3%).

Rape emerges as the type of violence that receives the most attention from professionals in most interventions, particularly those related to post-exposure or risk mitigation measures. This is the case for STI (56%), HIV (56%), and Hepatitis B (57%) prophylaxis, where rape rates exceed harassment by approximately 15 to 16% points. There is also a high rate of emergency contraception in cases of rape (68%).

On the other hand, harassment stands out in two specific procedures. In the collection of vaginal secretions, harassment accounts for 59% of records, surpassing rape, which was 37%. Similarly, in the context of abortion provided for by law, harassment (49%) is slightly more frequent than rape (45%). In blood and semen collection, the distribution between harassment (46% and 50%, respectively) and rape (49% and 44%, respectively) is more balanced.

## Discussion

The analysis of reports of sexual violence against children and adolescents living in peripheral regions of São Paulo suggests a scenario of vulnerability for public health. The trend observed between the occurrence and residence in peripheral administrative districts (*p* < .0001), added to the concentration of cases in neighborhoods such as Itaim Paulista, Tremembé-Jaçanã, and Campo Limpo, areas with a higher presence of subnormal agglomerations and densely populated collective housing, seems to be consistent with the literature on social determinants of violence in childhood [11]. Qualitative research in low- and middle-income countries has identified that unequal gender norms, poverty, and cultural context can act as factors that increase vulnerability to violence.

One possible interpretation focuses on the hypothesis that geographically vulnerable areas may have limited resources, which possibly increases material insecurity and parental stress. Additionally, the limited availability of support networks, such as specialized police stations and protection and health services, may reduce opportunities for reporting and resources allocated to mitigating violence against this population [12]. 

International evidence indicates that children living in socioeconomically disadvantaged neighborhoods with limited access to community support may face high risks of sexual violence and other forms of abuse. [12, 13] Neighborhoods with the highest incidence rates in São Paulo tend to be characterized by lower Human Development Index (HDI) scores and extensive pockets of poverty [10]. Although Brazil has implemented assistance programs for vulnerable families, persistent poverty seems to continue to influence the dynamics of family relationships.

To address this problem, economic strengthening strategies that seek to mitigate structural inequalities and reinforce protective factors in these areas are recommended. The profile of victims was predominantly female, with rape being the most prevalent form of violence. This pattern is similar to surveillance data and global epidemiological studies, in which girls and young women account for the majority of victims of sexual abuse [14]. The age distribution indicated a higher frequency of cases in the 11–14 age group. However, the rate of rape identified in early childhood is a relevant finding. Studies on the subject suggest that violence against younger children often involves known perpetrators, such as family members or acquaintances, which may be related with greater severity and possible long-term consequences. [14, 15]

In this study, no significant relationship was observed with respect to race/color. However, it should be noted that global evidence suggests that the intersection of low educational attainment, belonging to racial/ethnic minorities, and poverty can increase the vulnerability of children and adolescents [16]. It can be inferred that different forms of oppression tend to operate in an interconnected manner. In the context of sexual violence, vulnerability may not result from a single factor, but from the likely interaction of multiple forms of marginalization. Other social factors seem to contribute to the vulnerability of children in peripheral territories. Regarding pregnancy, the data obtained do not allow us to establish a direct causal relationship, since it is not possible to infer whether the pregnancy was a consequence of violence or preceded the abuse. In any case, sexual violence and pregnancy are sensitive issues of global magnitude, possibly influenced by sociocultural aspects such as male chauvinism and the sexual objectification of girls [17]. 

The presence of disability was also identified as a vulnerability factor, especially in cases of intellectual disability. Brazilian studies suggest that children and adolescents with intellectual disabilities may be subject to high rates of abuse and exploitation, possibly due to factors such as dependence, communication barriers, and difficulties in setting boundaries [18]. With regard to sexual orientation and gender identity, it was observed that, although heterosexuals account for higher absolute numbers, homosexual and bisexual people were more exposed to various forms of sexual violence. Analysis of gender identity suggested vulnerability for transgender women and men. Studies with the LGBTQ+ community indicate that transgender youth may face disproportionate rates of violence, often related with discrimination and minority stress [19]. 

The finding that the home was the most frequent location for most forms of violence corroborates the literature that points to the home as a space of risk, where power relations and family dynamics can manifest themselves. Studies conducted during the COVID-19 pandemic reinforced the hypothesis that isolation measures may have increased victims’ exposure to aggressors in the domestic environment [20]. Schools, identified as the second most common place for harassment, also emerge as a critical point, similar to international research that points to educational institutions as environments where harassment and bullying may be prevalent [21]. 

The identification of sexism as the predominant motivation suggests the structural nature of sexual violence against children in vulnerable areas. This finding is consistent with theories that position sexual violence as a possible mechanism for maintaining patriarchal structures. Female adolescents may be exposed to violence in romantic relationships, which would reinforce structural sexism, in which violence would operate as a means of domination and control. The use of physical force or beating as a predominant means highlights components of domination and intimidation. Threats, which are common in harassment, can act as psychological violence aimed at coercion. [22, 23] Research on trauma indicates that the threat of violence can trigger post-traumatic stress responses of similar intensity to physical aggression, with lasting effects [24]. 

Data on relationships point to a worrying context, in which family members and close friends are identified as the main perpetrators. The figure of the father as a frequent perpetrator in harassment and that of the boyfriend in rape among adolescents suggests that domestic and intimate partner violence may be recurrent. These findings are consistent with the literature that identifies family members and partners as common perpetrators [21–24]. The involvement of friends and acquaintances also signals vulnerability in close social networks. These results reinforce the importance of addressing violence in primary care consultations and in the school environment for the recognition and protection of children and adolescents. Reviews based on the WHO INSPIRE framework suggest strategies such as parenting programs, safe school environments, education for healthy relationships, cash transfers combined with training, and cognitive-behavioral therapy [25]. 

The predominance of male authors and adults (aged 25–59) seems to corroborate the understanding that sexual violence may be a behavior influenced by social gender norms, rather than a purely isolated impulse. [15, 16] The concentration in adulthood suggests that the power related with this stage of life may be instrumentalized in aggression. In contexts of vulnerability, alcohol consumption may be linked to coping with chronic adversity, possibly acting as a disinhibitor. However, simplistic causality is avoided; researchers suggest that alcohol may function as a contextual catalyst that interacts with preexisting gender norms. In such regions, the perception of impunity may be accentuated. Thus, multisectoral collaboration between families, schools, and the health sector is relevant to prevent harmful alcohol use and mitigate the effects of violence. [26, 27]

Healthcare has demonstrated a focus on post-rape procedures, such as prophylaxis and emergency contraception. This attention is understandable given the immediate severity and existing protocols. However, this raises questions about the scope of care for other forms of violence, such as harassment and exploitation. For peripheral populations, geographical distance and transportation costs can hinder access to specialized services. These factors, combined with stigma, can inhibit the search for care [28]. Sexual exploitation in vulnerable areas is often linked to extreme economic deprivation [29]. In addition, the possible normalization of some situations or fear and dependence on the exploiter can hinder detection. This requires proactive approaches and training of professionals to identify indirect signs [30]. Phenomena such as child pornography and harassment of people with disabilities may reflect determinants such as failures in protection [26, 29]. 

Finally, a considerable amount of ignored or blank data was observed in the variables investigated. It was decided not to further analyze this data to avoid misinterpretation. These results may be due to factors such as gaps in training for completing notifications or difficulties in understanding the variables. According to scholars, this incompleteness affects the quality of information and the planning of public policies, and should be the subject of continuous analysis by health surveillance [31]. 

### Study limitations and advances in science

Because this study covered only a specific period, it was not possible to establish cause-and-effect relationships or identify clear associations, as there were no non-case groups with which to determine the incidence risk. In addition, sexual violence is widely underreported, especially in urban peripheries, which means that the available records presumably represent only a portion of the cases that occurred. The quality of the information depended on the completion of forms by health professionals, which was subject to incompleteness, inconsistencies, and variations in training. Tabnet itself provided aggregated data, which limited more detailed analyses and prevented more complex investigations. Relevant contextual factors, such as family dynamics, history of violence, access to services, and territorial vulnerabilities, were not captured by the system, restricting understanding of the phenomenon. The increase in late notifications, made after the collection period, may have generated an increase in data that could perhaps improve the findings in this study.

Despite the limitations mentioned, the study offers relevant contributions to the advancement of science by broadening the understanding of the profile of sexual violence against children and adolescents in the urban peripheries of São Paulo, a phenomenon that has historically been invisible and underreported. By systematizing information available on Tabnet/DataSUS, the research identified patterns, the most vulnerable groups, and recurring characteristics of the episodes, providing input for the improvement of public policies, epidemiological surveillance, and prevention strategies. The study also contributes to the debate on the quality of health information systems and reinforces the need for investments in team training, strengthening the protection network, and producing more accurate and territory-sensitive data. Thus, even with its methodological limitations, the investigation provides useful knowledge both for the formulation of interventions and to guide future research on sexual violence against children and adolescents in contexts of vulnerability.

## Conclusions

The study identified the main characteristics of sexual violence against children and adolescents in the outskirts of São Paulo, highlighting its high magnitude and severity. Rape was the most prevalent form, affecting mainly girls between 11 and 14 years of age, although there were also significant occurrences in early childhood. A high concentration of cases was observed in districts with high poverty rates, reinforcing the relationship between socioeconomic vulnerability and exposure to violence. Social markers such disability, sexual orientation, and gender identity indicated additional vulnerabilities. The violence occurred mainly in the domestic environment and was perpetrated by people close to the victims, with frequent use of force, threats, and, in some cases, alcohol. There was a higher concentration of institutional procedures in cases of rape, suggesting possible gaps in the response to other forms of sexual violence.

The findings demonstrate that sexual violence against children and adolescents in the outskirts of São Paulo is a complex phenomenon, deeply rooted in social and structural inequalities, and that it requires robust intersectoral policies, continuous improvement of reporting practices, and strengthening of the protection network.

## Data Availability

Further information can be obtained at (https:/osf.io/du2yb/) (english data) or requested from the corresponding author.

## Abbreviations

COVID-19: Coronavirus Disease 2019
CSE: Commercial Sexual Exploitation
DATASUS: Department of Informatics of the Unified Health System
HDI: Human Development Index
HIV: Human Immunodeficiency Virus
IGN: Ignored
INSPIRE: Implementation and enforcement of laws, Normative and social change, Safe environments, Parents and Provider support, Income and economic empowerment, Response and Education
LGBTQ+: Lesbian, Gay, Bisexual, Transgender, Queer and more
PTSD: Post-Traumatic Stress Disorder
SINAN: Notifiable Diseases Information System
STIs: Sexually Transmitted Infections
STROBE: Strengthening the Reporting of Observational studies in Epidemiology
UK: United Kingdom
UNICEF: United Nations Children’s Fund
WHO: World Health Organization
